# Statistical analysis plan for transfusion of modified whole blood *versus* blood components therapy in patients with severe trauma: randomized controlled trial - WEBSTER trial

**DOI:** 10.62675/2965-2774.20260116

**Published:** 2026-10-23

**Authors:** Alberto Federico García, Andrés Gempeler, Edgar-Yaset Caicedo, Juan Carlos Puyana, Carlos A. Ordóñez, Gustavo Adolfo Ospina-Tascón

**Affiliations:** 1 División de Cirugía de Trauma y Emergencias Departamento de Cirugía Fundación Valle del Lili Cali Colombia División de Cirugía de Trauma y Emergencias, Departamento de Cirugía, Fundación Valle del Lili - Cali, Colombia.; 2 Departamento de Cuidado Intensivo Fundación Valle del Lili Cali Colombia Departamento de Cuidado Intensivo, Fundación Valle del Lili - Cali, Colombia.; 3 Centro de Investigaciones Clínicas Fundación Valle del Lili Cali Colombia Centro de Investigaciones Clínicas, Fundación Valle del Lili - Cali, Colombia.; 4 Especialización en Cirugía General Universidad Icesi Cali Colombia Especialización en Cirugía General, Universidad Icesi - Cali, Colombia.; 5 Global Health, Division of Trauma and Surgery University of Pittsburgh Pittsburgh PA United States Global Health, Division of Trauma and Surgery, University of Pittsburgh - Pittsburgh, PA, United States.; 6 Translational Research Lab in Critical Care Medicine Universidad Icesi Cali Colombia Translational Research Lab in Critical Care Medicine, Universidad Icesi - Cali, Colombia.

**Keywords:** Blood transfusion, Shock, hemorrhagic, Trauma, Transfusion, Multiple organ failure

## Abstract

**Background:**

Hemostatic resuscitation with modified whole blood is an alternative to balanced blood-component therapy for trauma patients with hemorrhagic shock, with potential efficacy and logistical advantages. The WEBSTER trial was designed to compare these interventions in patients with trauma hemorrhagic shock.

**Methods:**

Single-center, open-label, parallel-group randomized clinical trial comparing whole blood with blood components in adults with trauma hemorrhagic shock.

**Primary outcome:**

hierarchical composite of all-cause 28-day mortality and SOFA score during the first 5 days after randomization. We will estimate treatment effects using a win-ratio analysis that prioritizes mortality. Primary analysis follows the intention-to-treat principle; secondary analyses in as-treated and per-protocol populations are planned. Secondary outcomes include mortality at additional time points, organ dysfunction metrics, coagulation parameters, transfusion requirements, and adverse events.

**Results:**

Primary outcome analyses will report win ratios, and secondary outcomes will be analyzed with hypothesis tests or regression time-to-event models; 95% confidence intervals will accompany all estimates. We do not plan formal adjustment for multiplicity because the outcomes share the same underlying construct. All outcomes will be interpreted in the context of the overall pattern of results.

**Conclusion:**

This statistical analysis plan prespecifies transparent methods to assess the effect of whole blood *versus* blood-component therapy in trauma patients. The hierarchical framework, complementary sensitivity analyses and comprehensive interpretation are intended to provide a robust and clinically meaningful assessment of whole blood effects.

## BACKGROUND

The use of blood components (BC) is a key element in trauma patient management, where timely intervention is essential for correcting trauma-induced coagulopathy and improving short-term outcomes.^(1,2)^A transfusion strategy based on a 1:1:1 ratio of red blood cells, plasma, and platelets – designed to reflect physiological proportions – has been associated with improved survival and better hemorrhage control.^(3-5)^ However, significant technical and logistical challenges hinder widespread adoption, making routine implementation difficult in many trauma care settings.^(6)^

Whole blood (WB) transfusion, reintroduced by the U.S. military during the Iraq and Afghanistan wars, has since been incorporated into select civilian trauma centers.^(7)^ Advocates propose that WB provides a more physiologically balanced resuscitation, simplifies transfusion logistics, and enhances hemostatic properties compared to BC.^(8,9)^ Despite these theoretical benefits and a favorable safety profile, observational studies and systematic reviews have reported mixed findings, and strong clinical trial evidence supporting its widespread use is warranted.^(10,11)^

This article describes the statistical analysis plan for the WEBSTER trial to prevent post-hoc analysis bias and selective reporting bias.

### Study objectives

#### Primary objective

To assess the effects of hemostatic resuscitation with modified WB compared to BC on mortality and organ dysfunction in patients with trauma and hemorrhagic shock.

#### Secondary objectives

The trial will also assess the effects of WB compared with BC on secondary outcomes, including all-cause mortality within the first 6 hours, 24 hours, and at 28 days; incidence of multiple organ dysfunction; incidence and correction of trauma-induced coagulopathy; and incidence of serious adverse events. Tertiary outcomes (exploratory) are also considered and are listed in the Outcomes section.

## Overview of study design

### Trial design

The WEBSTER Trial is an open-label, single-center, parallel-group, superiority randomized controlled trial with two arms: modified whole blood *versus* BC transfusion, with a 1:1 allocation ratio. The trial protocol has been published^(12)^ and is registered in ClinicalTrials.gov (NCT05634109).

### Study population

Adult patients with blunt or penetrating trauma requiring transfusion for hemorrhagic shock at the emergency department within 4 hours of injury, with transfusion need defined by ABC score ≥ 2 or physician discretion, and concurrent availability of both whole blood and BC at randomization. Main exclusion criteria include pre-hospital surgery, catastrophic brain injury, in-extremis status, burns > 20% total body surface area, cardiopulmonary resuscitation before Emergency Department (ED) arrival or for longer than > 5 minutes prior to randomization, prior transfusion of more than one red blood cell unit, pregnancy, incarceration, and pre-existing do-not-resuscitate order. The published protocol describes the full eligibility criteria.^(12)^

## Randomization

This trial uses a 1:1 allocation ratio, achieved by randomization with permuted blocks of sizes 4 and 6. We generated the randomization sequence using the ‘blockrand’ and ‘randomizr’ packages in RStudio and uploaded it to REDCap. Allocation is concealed from and inaccessible to study investigators. Initially, we planned a stratified randomization based on the presence of moderate or severe traumatic brain injury (TBI) because of its impact on trauma mortality;^(13)^ nevertheless, TBI severity is difficult to assess in the emergency setting, which led to frequent patient misclassification during the study pilot. Therefore, after obtaining Institutional Review Board (IRB) approval, we randomized without stratification.

The participant’s assigned group is revealed only after the Emergency Room (ER) physician assesses the eligibility criteria and contacts the blood bank. Blood bank staff then log into REDCap and randomize using a web-based tool. This process does not interfere with clinical care or procedures in the ER or blood bank.

## Study interventions

Participants are allocated to either WB or BC via computer randomization at the blood bank upon request of a trauma transfusion package and confirmation of the patient’s eligibility from the ER. Transfusion of WB or BC follows standard institutional procedures guided by thromboelastography and conventional coagulation tests.

## Protocol deviations

### Adherence to protocol and protocol deviations

Protocol deviations will be reported as counts and proportions overall and by intervention groups. Major deviations that will determine analysis populations include deviations in participant recruitment (errors in applying eligibility criteria) and deviations from assigned interventions.

### Post-randomization exclusions

Given the trial’s emergency setting, post-randomization exclusions are expected at 10% to 20%, as reported in similar studies. These exclusions reflect missing inclusion criteria or missed exclusion criteria before randomization. According to Fergusson et al., acceptable post-randomization exclusions from the intention-to-treat (ITT) population include ineligible patients mistakenly included in the trial.^(14)^ So that the decision to remove these patients is not influenced by events after randomization and remains unbiased, we followed their recommendation to assess deviations regarding unfulfillment of eligibility criteria under blinding to treatment allocation and outcome. Two independent researchers will perform this task and assess the risk of bias or random error for each case, as the validity of excluding patients from all analyses or including them in the ITT (and as-treated) analysis depends primarily on the unfulfilled criterion. A third investigator will resolve discrepancies. This process will be performed in duplicate upon interim analysis and study completion. To ensure reproducibility, we will report all judgments and reasons for post-randomization exclusions with our results.

## Interim analysis

We planned an interim analysis upon completion of 50% of the calculated sample size for ITT analysis. We conducted the interim analysis while intervention assignment remained blinded after recruiting 126 patients, covering primary and safety outcomes only. Only the independent Data Safety Monitoring Board (DSMB) had access to the results. The DSMB comprised two experienced trauma surgeons and trialists and one senior statistician, who reviewed blinded safety outcomes and found no basis for unblinding or early termination.

## Sample size calculation and rationale

Feasibility considerations guided the sample size, with an estimated enrollment of 220 patients over 2 years, based on study-site data on the frequency of eligible patients in previous years. Simulations to assess the statistical power for detection of a clinically meaningful difference using the win ratio for the hierarchical composite outcome of death and Sequential Organ Failure Assessment (SOFA) score used an expected mortality of 21.8% and a SOFA score difference between days 1 and 5 of -1.38 (standard deviation = 3.02) under the standard of care. These values were derived from retrospective data from the study site and from a randomized controlled trial on hypertonic saline conducted at our institution in recent years, respectively.^(15)^ With 220 patients, a win ratio analysis would achieve > 80% power to detect absolute differences of 4% in mortality and 1.5 points in SOFA score between groups, as well as for different combinations of mortality and SOFA differences, as described in table 1.

**Table 1:** Empirical power of win-ratio analysis for different combinations of 28-day mortality and SOFA score differences between groups

Absolute difference in 28-day mortality (%)	SOFA score difference	Empirical power of win-ratio (%)
-2	-1	‘43
	-1.5	71.5
	-2	90.8
	-2.5	97.8
-3	-1	48
	-1.5	78.6
	-2	92.8
	-2.5	98.9
-4	-1	58
	-1.5	82.3
	-2	95.2
	-2.5	99

SOFA - Sequential Organ Failure Assessment.

Anticipating post-randomization exclusions due to eligibility assessment errors inherent to an emergency-setting trial, expected at a rate between 10% and 20%, we will need to randomize between 242 and 264 participants to ensure adequate statistical power for the primary analysis. Recruitment will thus stop when we complete 220 patients in the ITT population.

## Analysis populations

### Primary analysis: intention-to-treat population

The primary analysis will follow the ITT principle. This may include participants with exclusion criteria not identified before randomization but will not include participants randomized without meeting the inclusion criteria. In the ITT analysis, patients will be analyzed as part of the group allocated by randomization, regardless of non-adherence to the intended intervention.

### Secondary analyses: as-treated and per-protocol populations

Secondary analyses will be conducted in as-treated and per-protocol populations: the as-treated analysis will determine participants’ group according to the intervention received, despite differences from the randomized allocation, and will include participants despite other major protocol deviations. Any patient who received at least one unit of WB after randomization will be analyzed as part of the WB group in as-treated analysis. Per-protocol analysis will determine participants’ group according to the intervention received despite randomization in the same way as as-treated analysis but will exclude participants with other protocol deviations.

Figure 1 depicts the CONSORT flow diagram illustrating the composition of the primary and secondary analysis populations.

**Figure 1:**
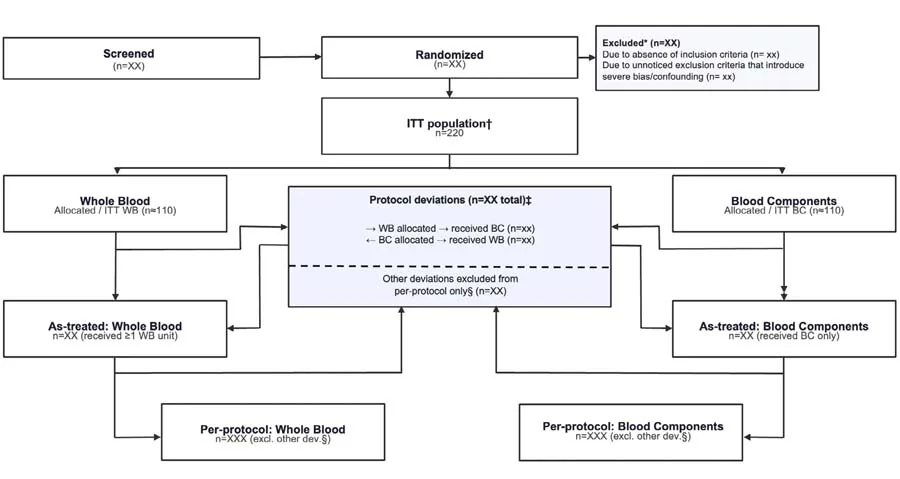
Flow diagram of trial screening, randomization and confirmation of primary and secondary analysis populations.

ITT - intention-to-treat population; WB - whole blood; BC - blood components

* Post-randomization exclusions (errors during eligibility screening inherent to emergency trial setting) adjudicated by two investigators blinded to treatment allocation; sum may exceed total as patients may meet > 1 criterion; † ITT - intention-to-treat population; all 220 randomized patients analyzed by allocation regardless of adherence; ‡ Protocol deviation box shows crossovers (patients receiving the opposite treatment) and other major deviations. Crossover patients are included in both as-treated and per-protocol analyses under the group corresponding to the treatment received. Patients with other deviations are included in as-treated but excluded from per-protocol analysis; § other deviations (non-crossover): e.g., post-randomization discovery of eligibility errors not excluded from the intention-to-treat population; non-adherence when the patient received both whole blood and blood components not contemplated in the trial protocol.

## Outcomes

### Interpretation framework

We will interpret consistent findings across analyses as supportive evidence of a treatment effect, while interpreting discordant findings cautiously.

Because these analyses address a single primary clinical question rather than multiple independent hypotheses, we will not formally adjust for multiplicity. Interpretation will emphasize the direction, magnitude, and consistency of effects rather than isolated statistical significance. We pre-specified secondary outcomes to formally evaluate clinically relevant consequences of the intervention and provide explanatory results. We will interpret these outcomes inferentially, focusing on effect estimates and confidence intervals. We will consider all tertiary outcomes exploratory and will not adjust for multiple comparisons in these analyses.

### Primary outcome

Hierarchical composite outcome of all-cause mortality at 28 days post-randomization and SOFA score progression in the 5 days after randomization. The hierarchical outcome requires defining wins, losses, and ties within pairs of participants from different groups. The win ratio will be calculated by comparing all participants in the whole blood group with all participants in the BC group. A win ratio > 1 indicates a net clinical benefit for the WB group. For each pair of participants (one from each group), outcomes are compared hierarchically as follows:

**Level 1 - Mortality at 28 days**

- If only one participant in the pair dies within 28 days, the surviving participant’s group receives a “win”.
- If both participants die, the comparison is considered a tie.
- If both participants survive, proceed to Level 2.

**Level 2 - SOFA score change (Day 1 to Day 5)**

- Calculate ΔSOFA = SOFA day 1 - SOFA day 5 for each participant (positive values indicate improvement), unless groups differ in baseline SOFA day 1 scores.
- Among pairs where both participants survive, the participant with the larger ΔSOFA (greater reduction in organ dysfunction) receives a “win” for that participant’s group.
- If ΔSOFA does not differ by at least 1 point, the comparison is considered a “tie”.
- If day 1 SOFA scores are different between groups -suggestive of risk of bias due to a larger reduction in patients with higher scores-, this level will compare SOFA scores at day 5 between pairs. Within a pair of survivors, the participant’s group with the lowest SOFA score at day 5 will receive a win.

### Pre-specified sensitivity analyses

During development of this statistical analysis plan, we identified a potential limitation of the protocol-specified outcome: measuring SOFA change only between days 1 and 5, or comparing only at day 5, may not capture peak organ dysfunction that develops and resolves between these measurement time points. Patients may experience clinically significant organ failure on days 2, 3, or 4 that improves by day 5, resulting in similar ΔSOFA or similar SOFA scores between groups despite substantially differential morbidity burden. To address this concern and ensure robust assessment of the intervention’s effect on organ dysfunction, we planned the following *a priori* sensitivity analysis:

**Modified Level 2 - Maximum SOFA score (Days 1 - 5)**

- Identify the maximum (highest) SOFA score recorded on any day from day 1 through day 5 for each participant.
- Among pairs where both participants survive 28 days, the participant with the lower maximum SOFA score receives a “win” for the participant’s group.
- If maximum SOFA score does not differ by at least 1 point, the comparison is considered a “tie”.

Given the known limitations of the win ratio in the presence of a large number of ties, we will conduct a sensitivity analysis using the win odds method as proposed by Brunner et al.^(16)^ The win odds is calculated as the total number of wins plus half the total number of ties, divided by the total number of losses plus half the total number of ties. This retains all pairwise information (does not exclude tied comparisons) by incorporating ties into both the numerator and denominator, reducing the potential for overestimation of treatment effect when a substantial proportion of pairs are tied. Interpretation of the results will prioritize consistency in the direction of the win estimates.

### Secondary outcomes

***Mortality***

Incidence of all-cause mortality and specific-cause mortality at 6-hours, 24-hours and 28 days post-randomization. If a difference in mortality between groups greater than 5% is detected, we will analyze all other secondary outcomes using a competing-risks approach with death.

***Multiple organ dysfunction***

Two approaches are used to ensure that any clinically relevant effects of the intervention on organ dysfunction will be detected:

- Incidence of multiple organ dysfunction (MOD) in the first 5 days after randomization.
- Highest SOFA scores in the first 5 days after randomization.

MOD is defined as SOFA score ≥ 2 points in one system or ≥ 1 in two or more systems on the same day. SOFA subscores will be reported to identify effects in particular systems that could be affected by the interventions, including the coagulation subscore.

***Serious adverse events***

We will compare the incidence and number of serious adverse events (SAEs) between groups overall and according to the potential relationship determined by the adjudication committee. Serious adverse events are defined according to Good Clinical Practice Guidelines, and our study protocol describes this process in detail.

***Coagulopathy***

Coagulopathy includes hypocoagulability and fibrinolytic coagulopathy. We will define hypocoagulability as thromboelastographic (TEG) parameters R > 10 min, K > 2.5 min, α < 65º, MA < 55mm, or conventional clotting tests showing INR > 1.2, platelets < 100.000/mm3, or fibrinogen < 2g/L. Fibrinolytic coagulopathy will be defined as LY-30 > 8%. We will compare groups regarding:

- Prevalence of coagulopathy at 6 hours and 12 hours after randomization.
- Incidence of hypocoagulability or fibrinolytic coagulopathy in the first 24 hours after randomization. For this cumulative incidence, we will not include patients with coagulopathy before randomization.
- Difference in SOFA coagulation sub score at days 1 and 5.

Thromboelastographic parameters, clotting times, and fibrinogen trends during the first 24 hours post-randomization will be assessed graphically using trajectory plots for individual participants by intervention group.

## Blood transfusion requirements during the first 24 hours

Difference in the number of units of WB or BC between groups. Comparisons will follow the following definitions: 1 unit of whole blood = 1 unit of packed red blood cells; 1 unit of whole blood = 1 unit of plasma; and 1 unit of whole blood = 1 unit of platelets.

### Length of hospital stay

Difference in hospital-free days at day 28 after randomization between WB and BC.

### Tertiary outcomes

- Vasopressor-free days at day 28 after randomization.
- Mechanical ventilation-free days at day 28 after randomization.
- Renal replacement therapy-free days at day 28 after randomization.
- Proportion of participants requiring damage control surgery in the first 28 days after randomization.
- Number of surgical procedures after index surgery or until cavity closure up to day 28 after randomization.
- Resuscitation fluids volume in the first 24 hours after randomization.
- Serum lactate in the first 3, 6, 12 and 24 hours after randomization.
- Hemoglobin and hematocrit progression in the first 24 hours after randomization.
- Leucocytes progression in the first 24 hours after randomization.
- Platelets progression in the first 24 hours after randomization.
- pH progression in the first 24 hours after randomization.
- Base excess progression in the first 24 hours after randomization.
- Progression of sodium, potassium, and ionic calcium in the first 24 hours after randomization.

## Statistical analyses

### General principles

Statistical analyses will be conducted in RStudio (R, Posit). The Win Ratio will be calculated using the WINS package. We will report statistical analyses using summary tables (Supplementary Material), figures, and data listings. We will summarize continuous variables as median (interquartile range) and range (min-max). Qualitative variables will be expressed as counts and proportions.

### Confidence intervals and p values

All effect estimates will be reported with 95% confidence intervals (95%CIs). Hypothesis tests will be two-tailed with a 5% significance level (α = 0.05). The win ratio and its 95%CI will be estimated on the log scale using a U-statistic-based variance estimator that accounts for the covariance between wins and losses,^(17)^ then back-transformed to the original scale. We will derive the corresponding two-sided p-value from the same variance estimator. The win odds 95% CI will be estimated using the same log-scaled U-statistic–based variance estimator accounting for the covariance among wins, losses, and ties.

## Data quality verification and database lock

Automated range and consistency checks are programmed within REDCap. Discrepancies identified during data review are raised as queries to trial coordinators, documented, and resolved as part of ongoing monitoring that verifies accuracy of all variables in the database. REDCap maintains an audit trail of all data changes.

The trial database will be locked upon completion of the 220-sample size for the ITT population, which implies: completion of data entry, completion of data monitoring, and resolution of all open queries. The principal investigator and data manager will authorize the database lock before statistical analysis. A clean analysis dataset will be exported from REDCap following database lock. The dataset will be version-controlled and frozen prior to unblinding.

## Handling of missing data

Missing SOFA scores will be handled using pre-specified interpolation rules as described in the published protocol.

For all secondary outcome variables, we will use complete-case analysis as the primary approach, provided missingness does not exceed pre-specified thresholds for any given outcome (> 5% missingness overall or a difference >3% in missing data between groups; excluding missingness attributable to death). If exceeded, we will conduct multiple imputation (MI) using the multivariate imputation by chained equations (MICE) algorithm with 50 imputations, with predictive mean matching for continuous variables and logistic regression for binary variables. The imputation model will include baseline characteristics, SOFA component and total scores at all available time points, treatment assignment, and survival status. We will assess convergence by inspecting trace plots across iterations before pooling. We will obtain pooled estimates using Rubin’s rules. We will report a sensitivity analysis comparing complete-case and MI results to assess the robustness of imputation assumptions.

## Analysis methods

### Baseline characteristics and treatments received

We will describe the baseline clinical and paraclinical characteristics of participants and the interventions received during the trial for the three analysis populations.

## Outcomes analysis plan

**Composite hierarchical outcome of death or multiple organ dysfunction (primary outcome):** after interpolation of SOFA scores as described in our published protocol, primary outcomes will be assessed in an “all-against-all” win ratio analysis that will report win ratio (WR) (total wins in intervention groups divided by total wins in control group) accompanied by 95% confidence interval.

### Secondary outcomes

- **Mortality:** we will assess differences in mortality with the chi^2^test and report the risk ratio (RR) with 95%CI. Our published protocol pre-specified that we would apply competing risks analyses conditionally if observed mortality differed by more than 5% between groups. Following reviewer feedback and methodological review, we recognized that competing risks are present whenever death can preclude the occurrence of an outcome, independently of the magnitude of between-group mortality differences. Accordingly, we will compare dichotomous secondary outcomes between groups using chi-squared tests or Fisher’s exact test, as appropriate, report risk differences and 95%CI, and report Fine-Gray subdistribution hazard models as sensitivity analyses for all dichotomous outcomes where death represents a competing risk. For all days-free outcomes, death before day 28 will be assigned zero free days, which inherently accounts for the competing nature of death for these endpoints.
- **Multiple organ dysfunction:** we will assess differences in the incidence of MOD during the first 5 days after randomization using the chi^2^test. Differences between groups in the highest SOFA score recorded will be assessed with the Student t test or the Wilcoxon-Mann-Whitney U test, as appropriate.
- **Serious adverse events:** differences in the incidence of SAE will be assessed with a chi^2^test in the absence of competing risks.
- **Coagulopathy:** differences in the incidence of coagulopathy will be assessed with a chi^2^test in the absence of competing risks.
- **Blood transfusion requirements:** differences in transfusion requirements will be assessed with the Student *t* test or the Wilcoxon-Mann-Whitney U test, as appropriate based on data distribution.

If there is an imbalance in baseline prognostic characteristics between groups, a sensitivity analysis of the effect of intervention on secondary outcomes will be adjusted accordingly by use of a multivariable logistic regression model for dichotomous outcomes and a generalized linear model with Gaussian distribution and log-link function for quantitative outcomes, both following a purposeful variable selection process to control for confounding.^(18)^ All mock tables (for baseline characteristics, interventions received and outcomes), stratified by intervention groups for primary and secondary analysis populations are available in tables 1S - 4S (Supplementary Material).

### Subgroup analysis

With an exploratory purpose, the treatment effects will be assessed on primary and secondary outcomes according to the following subgroups: age (< and ≥ 50 years of age), time between the trauma and randomization according to terciles, time between admission and transfusion (< and ≥ 30 minutes), time for hemostatic resuscitation (< and ≥ 120 minutes), systolic blood pressure on admission (< and ≥ 90mmHg), lactate levels on admission (< and ≥ 4mmol/L), INR on admission (< and ≥ 1.8), ionic calcium on admission (< and ≥ 0.9mmol/L), TEG-MA parameter on admission (< and ≥ 55mm); and injury severity score (< and ≥ 25 points).

## CONCLUSION

This statistical analysis plan pre-specifies the analytic principles, outcome definitions, and statistical framework for analyzing and interpreting WEBSTER trial results. By prespecifying all primary, secondary, and exploratory analyses before database lock, this paper aims to safeguard the trial against data-driven analyses, selective reporting, and post hoc reinterpretation of results. The planned analyses are grounded in the intention-to-treat principle, with clearly defined estimands, missing-data handling, and prespecified approaches to sensitivity analyses. We paid special attention to the complex clinical context of hemorrhagic shock, including competing risks, to ensure that statistical methods align with the underlying pathophysiology and trial objectives.
