# Supplementary material for: Statistical analysis plan for transfusion of modified whole blood versus blood components therapy in patients with severe trauma: randomized controlled trial - WEBSTER trial
Source: Crit Care Sci. 2026 Oct 23;38:e20260116. doi: 10.62675/2965-2774.20260116 (PMC13690036; doi:10.62675/2965-2774.20260116)
Supplement: Supplementary file 1 [file 2965-2774-ccsci-38-e20260116-suppl.pdf]

Critical Care Science
https://doi.org/10.62675/2965-2774.20260116

SUPPLEMENTARY MATERIAL

Statistical analysis plan for transfusion of modified whole blood *versus* blood components therapy in patients with severe trauma: randomized controlled trial - WEBSTER trial

Alberto Federico García[1,2], Andrés Gempeler[3], Edgar-Yaset Caicedo[3,4], Juan Carlos Puyana[5], Carlos A. Ordóñez[1,2], Gustavo Adolfo Ospina-Tascón[2,6]

MOCK TABLES FOR WEBSTER TRIAL

Table 1S - Baseline characteristics of included participants by intervention group and analysis populations

Characteristic	Intention-to-treat set		Per-protocol set		As-treated set	
	Whole blood (n = XX)	Blood components (n = XX)	Whole blood (n = XX)	Blood components (n = XX)	Whole blood (n = XX)	Blood components (n = XX)
Demographics and presentation						
Age (years)	xx (xx)	xx (xx)	xx (xx)	xx (xx)	xx (xx)	xx (xx)
Male - n (%)	xx (xx)	xx (xx)	xx (xx)	xx (xx)	xx (xx)	xx (xx)
Referred from another institution - n (%)	xx (xx)	xx (xx)	xx (xx)	xx (xx)	xx (xx)	xx (xx)
Healthcare level of referring institution						
Level I	xx (xx)	xx (xx)	xx (xx)	xx (xx)	xx (xx)	xx (xx)
Level II	xx (xx)	xx (xx)	xx (xx)	xx (xx)	xx (xx)	xx (xx)
Level III	xx (xx)	xx (xx)	xx (xx)	xx (xx)	xx (xx)	xx (xx)
Not applicable	xx (xx)	xx (xx)	xx (xx)	xx (xx)	xx (xx)	xx (xx)
Pre-admission IV fluids (mL)	xx (xx)	xx (xx)	xx (xx)	xx (xx)	xx (xx)	xx (xx)
Trauma-to-door interval (min)	xx (xx)	xx (xx)	xx (xx)	xx (xx)	xx (xx)	xx (xx)
Trauma-to-randomization interval (min)	xx (xx)	xx (xx)	xx (xx)	xx (xx)	xx (xx)	xx (xx)
Intrahospital CPR - n (%)	xx (xx)	xx (xx)	xx (xx)	xx (xx)	xx (xx)	xx (xx)
Intrahospital CPR duration (min)	xx (xx)	xx (xx)	xx (xx)	xx (xx)	xx (xx)	xx (xx)
Vital signs at admission						
Systolic blood pressure (mmHg)	xx (xx)	xx (xx)	xx (xx)	xx (xx)	xx (xx)	xx (xx)
Diastolic blood pressure (mmHg)	xx (xx)	xx (xx)	xx (xx)	xx (xx)	xx (xx)	xx (xx)
Mean arterial pressure (mmHg)	xx (xx)	xx (xx)	xx (xx)	xx (xx)	xx (xx)	xx (xx)
Heart rate (bpm)	xx (xx)	xx (xx)	xx (xx)	xx (xx)	xx (xx)	xx (xx)
Respiratory rate (rpm)	xx (xx)	xx (xx)	xx (xx)	xx (xx)	xx (xx)	xx (xx)
Temperature (°C)	xx (xx)	xx (xx)	xx (xx)	xx (xx)	xx (xx)	xx (xx)

Continue...

This is an open access article under the CC BY license (https://creativecommons.org/licenses/by/4.0/).

...continuation

Glasgow Coma Scale	xx (xx)	xx (xx)	xx (xx)	xx (xx)	xx (xx)	xx (xx)
Capillary refill time (seconds)	xx (xx)	xx (xx)	xx (xx)	xx (xx)	xx (xx)	xx (xx)
Trauma mechanism - n (%)						
Penetrating	xx (xx)	xx (xx)	xx (xx)	xx (xx)	xx (xx)	xx (xx)
Type of penetrating trauma - n (%)						
Firearm	xx (xx)	xx (xx)	xx (xx)	xx (xx)	xx (xx)	xx (xx)
Sharp weapon	xx (xx)	xx (xx)	xx (xx)	xx (xx)	xx (xx)	xx (xx)
Impalement	xx (xx)	xx (xx)	xx (xx)	xx (xx)	xx (xx)	xx (xx)
Not applicable	xx (xx)	xx (xx)	xx (xx)	xx (xx)	xx (xx)	xx (xx)
Blunt	xx (xx)	xx (xx)	xx (xx)	xx (xx)	xx (xx)	xx (xx)
Type of blunt trauma - n (%)						
Fall	xx (xx)	xx (xx)	xx (xx)	xx (xx)	xx (xx)	xx (xx)
Traffic accident (car)	xx (xx)	xx (xx)	xx (xx)	xx (xx)	xx (xx)	xx (xx)
Traffic accident (motorcycle)	xx (xx)	xx (xx)	xx (xx)	xx (xx)	xx (xx)	xx (xx)
Traffic accident (pedestrian)	xx (xx)	xx (xx)	xx (xx)	xx (xx)	xx (xx)	xx (xx)
Aggression	xx (xx)	xx (xx)	xx (xx)	xx (xx)	xx (xx)	xx (xx)
Not applicable	xx (xx)	xx (xx)	xx (xx)	xx (xx)	xx (xx)	xx (xx)
e-FAST - n (%)						
Negative	xx (xx)	xx (xx)	xx (xx)	xx (xx)	xx (xx)	xx (xx)
Positive	xx (xx)	xx (xx)	xx (xx)	xx (xx)	xx (xx)	xx (xx)
Not performed	xx (xx)	xx (xx)	xx (xx)	xx (xx)	xx (xx)	xx (xx)
Haematology						
Hemoglobin (g/dL)	xx (xx)	xx (xx)	xx (xx)	xx (xx)	xx (xx)	xx (xx)
Hematocrit (%)'	xx (xx)	xx (xx)	xx (xx)	xx (xx)	xx (xx)	xx (xx)
Platelets ($\times 10^3/\mu L$)	xx (xx)	xx (xx)	xx (xx)	xx (xx)	xx (xx)	xx (xx)
Blood group - n (%)						
A (+)	xx (xx)	xx (xx)	xx (xx)	xx (xx)	xx (xx)	xx (xx)
A (−)	xx (xx)	xx (xx)	xx (xx)	xx (xx)	xx (xx)	xx (xx)
B (+)	xx (xx)	xx (xx)	xx (xx)	xx (xx)	xx (xx)	xx (xx)
B (−)	xx (xx)	xx (xx)	xx (xx)	xx (xx)	xx (xx)	xx (xx)
AB (+)	xx (xx)	xx (xx)	xx (xx)	xx (xx)	xx (xx)	xx (xx)
AB (−)	xx (xx)	xx (xx)	xx (xx)	xx (xx)	xx (xx)	xx (xx)
O (+)	xx (xx)	xx (xx)	xx (xx)	xx (xx)	xx (xx)	xx (xx)
O (−)	xx (xx)	xx (xx)	xx (xx)	xx (xx)	xx (xx)	xx (xx)
Blood gas and chemistry						
pH	xx (xx)	xx (xx)	xx (xx)	xx (xx)	xx (xx)	xx (xx)
$PaCO_2$ (mmHg)	xx (xx)	xx (xx)	xx (xx)	xx (xx)	xx (xx)	xx (xx)
HCO_3 (mmol/L)	xx (xx)	xx (xx)	xx (xx)	xx (xx)	xx (xx)	xx (xx)
Base excess (mmol/L)	xx (xx)	xx (xx)	xx (xx)	xx (xx)	xx (xx)	xx (xx)
PaO_2:FiO_2	xx (xx)	xx (xx)	xx (xx)	xx (xx)	xx (xx)	xx (xx)

Continue...

...continuation

Lactate (mmol/L)	xx (xx)	xx (xx)	xx (xx)	xx (xx)	xx (xx)	xx (xx)
Creatinine (mg/dL)	xx (xx)	xx (xx)	xx (xx)	xx (xx)	xx (xx)	xx (xx)
Blood urea nitrogen (mg/dL)	xx (xx)	xx (xx)	xx (xx)	xx (xx)	xx (xx)	xx (xx)
Ionic calcium (mmol/L)	xx (xx)	xx (xx)	xx (xx)	xx (xx)	xx (xx)	xx (xx)
Conventional coagulation						
Prothrombin time (sec)	xx (xx)	xx (xx)	xx (xx)	xx (xx)	xx (xx)	xx (xx)
Thromboplastin time (sec)	xx (xx)	xx (xx)	xx (xx)	xx (xx)	xx (xx)	xx (xx)
INR	xx (xx)	xx (xx)	xx (xx)	xx (xx)	xx (xx)	xx (xx)
Fibrinogen (mg/dL)	xx (xx)	xx (xx)	xx (xx)	xx (xx)	xx (xx)	xx (xx)
Thromboelastography						
R-TEG time (min)	xx (xx)	xx (xx)	xx (xx)	xx (xx)	xx (xx)	xx (xx)
K-TEG time (min)	xx (xx)	xx (xx)	xx (xx)	xx (xx)	xx (xx)	xx (xx)
α-angle (degrees)	xx (xx)	xx (xx)	xx (xx)	xx (xx)	xx (xx)	xx (xx)
MA (mm)	xx (xx)	xx (xx)	xx (xx)	xx (xx)	xx (xx)	xx (xx)
LY-30 (%)	xx (xx)	xx (xx)	xx (xx)	xx (xx)	xx (xx)	xx (xx)
Injury scoring						
Abbreviated Injury Score - median (IQR)						
Head	xx (xx)	xx (xx)	xx (xx)	xx (xx)	xx (xx)	xx (xx)
Abdomen	xx (xx)	xx (xx)	xx (xx)	xx (xx)	xx (xx)	xx (xx)
Thorax	xx (xx)	xx (xx)	xx (xx)	xx (xx)	xx (xx)	xx (xx)
Limbs	xx (xx)	xx (xx)	xx (xx)	xx (xx)	xx (xx)	xx (xx)
External	xx (xx)	xx (xx)	xx (xx)	xx (xx)	xx (xx)	xx (xx)
Injury Severity Score	xx (xx)	xx (xx)	xx (xx)	xx (xx)	xx (xx)	xx (xx)
New Injury Severity Score	xx (xx)	xx (xx)	xx (xx)	xx (xx)	xx (xx)	xx (xx)
Revised Trauma Score	xx (xx)	xx (xx)	xx (xx)	xx (xx)	xx (xx)	xx (xx)
Activation of massive transfusion protocol						
ABC Score ≥ 2 - n (%)	xx (xx)	xx (xx)	xx (xx)	xx (xx)	xx (xx)	xx (xx)
ABC Score value (0 - 4)	xx (xx)	xx (xx)	xx (xx)	xx (xx)	xx (xx)	xx (xx)
Shock Index	xx (xx)	xx (xx)	xx (xx)	xx (xx)	xx (xx)	xx (xx)

IV - intravenous; CPR - cardiopulmonary resuscitation; e-FAST - extended focused assessment with sonography in trauma; $PaCO_2$ - partial pressure of arterial carbon dioxide; HCO_3 - bicarbonate; FiO_2 fraction of inspired oxygen; INR - international normalized ratio; TEG - thromboelastography; MA - maximum amplitude; IQR - interquartile range; ABC - Assessment of Blood Consumption. Continuous variables assessed for normality by visual inspection of histograms and D'Agostino-Pearson test; normally distributed variables reported as mean (± standard deviation). No formal hypothesis testing for baseline characteristics; p values not reported. We note any apparent imbalances narratively in the Results. Data are presented as median (interquartile range) for continuous variables and n (%) for categorical variables, unless otherwise specified.

Table 2S - Interventions received by group and analysis population

Variable	Intention-to-treat set			Per-protocol set			As-treated set		
	Whole blood (n = xx)	Blood components (n = xx)	p value*	Whole blood (n = xx)	Blood components (n = xx)	p value*	Whole blood (n = xx)	Blood components (n = xx)	p value*
Transfusion timing									
Trauma-to-randomization interval (minute)	xx (xx - xx)	xx (xx - xx)	x.xxx	xx (xx–xx)	xx (xx–xx)	x.xxx	xx (xx–xx)	xx (xx–xx)	x.xxx
Trauma-to-first-transfusion interval (minute)	xx (xx - xx)	xx (xx - xx)	x.xxx	xx (xx–xx)	xx (xx–xx)	x.xxx	xx (xx–xx)	xx (xx–xx)	x.xxx
Transfusion duration (minute)	xx (xx - xx)	xx (xx - xx)	x.xxx	xx (xx–xx)	xx (xx–xx)	x.xxx	xx (xx–xx)	xx (xx–xx)	x.xxx
Study intervention products - cumulative 24 hours†									
Modified whole blood units, n	xx (xx - xx)	xx (xx - xx)	x.xxx	xx (xx–xx)	xx (xx–xx)	x.xxx	xx (xx–xx)	xx (xx–xx)	x.xxx
Modified whole blood volume (mL)	xx (xx - xx)	xx (xx - xx)	x.xxx	xx (xx - xx)	xx (xx - xx)	x.xxx	xx (xx - xx)	xx (xx - xx)	x.xxx
Blood component products - cumulative 24 hours†									
pRBC units, n	xx (xx - xx)	xx (xx - xx)	x.xxx	xx (xx - xx)	xx (xx - xx)	x.xxx	xx (xx - xx)	xx (xx - xx)	x.xxx
pRBC volume (mL)	xx (xx - xx)	xx (xx - xx)	x.xxx	xx (xx - xx)	xx (xx - xx)	x.xxx	xx (xx - xx)	xx (xx - xx)	x.xxx
Plasma units, n	xx (xx - xx)	xx (xx - xx)	x.xxx	xx (xx - xx)	xx (xx - xx)	x.xxx	xx (xx - xx)	xx (xx - xx)	x.xxx
Plasma volume (mL)	xx (xx - xx)	xx (xx - xx)	x.xxx	xx (xx - xx)	xx (xx - xx)	x.xxx	xx (xx - xx)	xx (xx - xx)	x.xxx
Platelets units, n	xx (xx - xx)	xx (xx - xx)	x.xxx	xx (xx - xx)	xx (xx - xx)	x.xxx	xx (xx - xx)	xx (xx - xx)	x.xxx
Platelets volume (mL)	xx (xx - xx)	xx (xx - xx)	x.xxx	xx (xx - xx)	xx (xx - xx)	x.xxx	xx (xx - xx)	xx (xx - xx)	x.xxx
Cryoprecipitate units, n	xx (xx - xx)	xx (xx - xx)	x.xxx	xx (xx - xx)	xx (xx - xx)	x.xxx	xx (xx - xx)	xx (xx - xx)	x.xxx
Cryoprecipitate volume (mL)	xx (xx - xx)	xx (xx - xx)	x.xxx	xx (xx - xx)	xx (xx - xx)	x.xxx	xx (xx - xx)	xx (xx - xx)	x.xxx
Cell Saver volume (mL)	xx (xx - xx)	xx (xx - xx)	x.xxx	xx (xx - xx)	xx (xx - xx)	x.xxx	xx (xx - xx)	xx (xx - xx)	x.xxx
Whole-blood equivalent units - cumulative 24 hours‡									
Total WB-equivalent units (WB + pRBC + plasma + platelets)	xx (xx - xx)	xx (xx - xx)	x.xxx	xx (xx - xx)	xx (xx - xx)	x.xxx	xx (xx - xx)	xx (xx - xx)	x.xxx
WB-equivalent ratio (WB:total)	xx (xx - xx)	xx (xx - xx)	x.xxx	xx (xx - xx)	xx (xx - xx)	x.xxx	xx (xx - xx)	xx (xx - xx)	x.xxx
Resuscitation fluids - cumulative 24 hours									
Crystalloids (mL)	xx (xx - xx)	xx (xx - xx)	x.xxx	xx (xx - xx)	xx (xx - xx)	x.xxx	xx (xx - xx)	xx (xx - xx)	x.xxx
Iso-oncotic albumin (mL)	xx (xx - xx)	xx (xx - xx)	x.xxx	xx (xx - xx)	xx (xx - xx)	x.xxx	xx (xx - xx)	xx (xx - xx)	x.xxx
Urine output at 24 hours (mL)	xx (xx - xx)	xx (xx - xx)	x.xxx	xx (xx - xx)	xx (xx - xx)	x.xxx	xx (xx - xx)	xx (xx - xx)	x.xxx
Fluid balance at 24 hours (mL)	xx (xx - xx)	xx (xx - xx)	x.xxx	xx (xx - xx)	xx (xx - xx)	x.xxx	xx (xx - xx)	xx (xx - xx)	x.xxx
Vasopressor support at 24 hours									
Any vasopressor	xx (xx)	xx (xx)	x.xxx	xx (xx)	xx (xx)	x.xxx	xx (xx)	xx (xx)	x.xxx
Norepinephrine - n (%)	xx (xx)	xx (xx)	x.xxx	xx (xx)	xx (xx)	x.xxx	xx (xx)	xx (xx)	x.xxx
Norepinephrine dose (µg/kg/minute)	xx (xx - xx)	xx (xx - xx)	-	xx (xx - xx)	xx (xx - xx)	-	xx (xx - xx)	xx (xx - xx)	-
Vasopressin - n (%)	xx (xx)	xx (xx)	x.xxx	xx (xx)	xx (xx)	x.xxx	xx (xx)	xx (xx)	x.xxx
Vasopressin dose (units/hour)	xx (xx - xx)	xx (xx - xx)	-	xx (xx - xx)	xx (xx - xx)	-	xx (xx - xx)	xx (xx - xx)	-
Dobutamine - n (%)	xx (xx)	xx (xx)	x.xxx	xx (xx)	xx (xx)	x.xxx	xx (xx)	xx (xx)	x.xxx
Adrenaline - n (%)	xx (xx)	xx (xx)	x.xxx	xx (xx)	xx (xx)	x.xxx	xx (xx)	xx (xx)	x.xxx

Continue...

...continuation

Hemorrhage control (first 6 hours)									
Hemorrhage control performed - n (%)	xx (xx)	xx (xx)	x.xxx	xx (xx)	xx (xx)	x.xxx	xx (xx)	xx (xx)	x.xxx
Surgical control - n (%)	xx (xx)	xx (xx)	x.xxx	xx (xx)	xx (xx)	x.xxx	xx (xx)	xx (xx)	x.xxx
Laparotomy - n (%)	xx (xx)	xx (xx)	x.xxx	xx (xx)	xx (xx)	x.xxx	xx (xx)	xx (xx)	x.xxx
Thoracotomy - n (%)	xx (xx)	xx (xx)	x.xxx	xx (xx)	xx (xx)	x.xxx	xx (xx)	xx (xx)	x.xxx
Sternotomy - n (%)	xx (xx)	xx (xx)	x.xxx	xx (xx)	xx (xx)	x.xxx	xx (xx)	xx (xx)	x.xxx
Peripheral vascular exploration - n (%)	xx (xx)	xx (xx)	x.xxx	xx (xx)	xx (xx)	x.xxx	xx (xx)	xx (xx)	x.xxx
Damage control surgery - n (%)	xx (xx)	xx (xx)	x.xxx	xx (xx)	xx (xx)	x.xxx	xx (xx)	xx (xx)	x.xxx
Definitive cavity closure in index surgery - n (%)	xx (xx)	xx (xx)	x.xxx	xx (xx)	xx (xx)	x.xxx	xx (xx)	xx (xx)	x.xxx
Endovascular control (IR) - n (%)	xx (xx)	xx (xx)	x.xxx	xx (xx)	xx (xx)	x.xxx	xx (xx)	xx (xx)	x.xxx
Conservative management - n (%)	xx (xx)	xx (xx)	x.xxx	xx (xx)	xx (xx)	x.xxx	xx (xx)	xx (xx)	x.xxx
REBOA / aortic clamping - n (%)	xx (xx)	xx (xx)	x.xxx	xx (xx)	xx (xx)	x.xxx	xx (xx)	xx (xx)	x.xxx
Estimated intraoperative blood loss (mL)	xx (xx - xx)	xx (xx - xx)	-	xx (xx - xx)	xx (xx - xx)	-	xx (xx - xx)	xx (xx - xx)	-
Index surgery duration (minute)	xx (xx - xx)	xx (xx - xx)	-	xx (xx - xx)	xx (xx - xx)	-	xx (xx - xx)	xx (xx - xx)	-
Vital signs pre- and post-transfusion§									
SBP pre-transfusion (mmHg)	xx (xx - xx)	xx (xx - xx)	x.xxx	xx (xx - xx)	xx (xx - xx)	x.xxx	xx (xx - xx)	xx (xx - xx)	x.xxx
SBP post-transfusion (mmHg)	xx (xx - xx)	xx (xx - xx)	x.xxx	xx (xx - xx)	xx (xx - xx)	x.xxx	xx (xx - xx)	xx (xx - xx)	x.xxx
HR pre-transfusion (bpm)	xx (xx - xx)	xx (xx - xx)	x.xxx	xx (xx - xx)	xx (xx - xx)	x.xxx	xx (xx - xx)	xx (xx - xx)	x.xxx
HR post-transfusion (bpm)	xx (xx - xx)	xx (xx - xx)	x.xxx	xx (xx - xx)	xx (xx - xx)	x.xxx	xx (xx - xx)	xx (xx - xx)	x.xxx
MAP pre-transfusion (mmHg)	xx (xx - xx)	xx (xx - xx)	x.xxx	xx (xx - xx)	xx (xx - xx)	x.xxx	xx (xx - xx)	xx (xx - xx)	x.xxx
MAP post-transfusion (mmHg)	xx (xx - xx)	xx (xx - xx)	x.xxx	xx (xx - xx)	xx (xx - xx)	x.xxx	xx (xx - xx)	xx (xx - xx)	x.xxx
Temperature pre-transfusion (°C)	xx (xx - xx)	xx (xx - xx)	x.xxx	xx (xx - xx)	xx (xx - xx)	x.xxx	xx (xx - xx)	xx (xx - xx)	x.xxx
Temperature post-transfusion (°C)	xx (xx - xx)	xx (xx - xx)	x.xxx	xx (xx - xx)	xx (xx - xx)	x.xxx	xx (xx - xx)	xx (xx - xx)	x.xxx

pRBC - packed red blood cells; WB - whole blood; IR - interventional radiology; REBOA - resuscitative endovascular balloon occlusion of the aorta; SBP - systolic blood pressure; HR - heart rate; MAP - mean arterial pressure. * Two-tailed p-value; Mann-Whitney U test for quantitative variables, chi^2 for categorical. Significance threshold $\alpha = 0.05$; † blood products reported cumulatively at 24 hours post-randomization across all timepoints (3 hours, 6 hours, 12 hours, 24 hours). Crossover products (e.g., packed red blood cells/plasma/platelets given to WB-allocated patients after protocol completion, or whole blood given to blood cells-allocated patients) are included in the respective group totals; ‡ whole blood-equivalent units defined per statistical analysis plan conversion: 1 unit whole blood = 1 unit packed red blood cells = 1 unit plasma = 1 unit platelets. Whole blood-equivalent ratio = whole blood units received ÷ total whole blood-equivalent units; § pre- and post-transfusion vital signs recorded at time of intervention. - = exploratory variable; no formal hypothesis testing pre-specified. Data presented as median (interquartile range) for continuous variables and n (%) for categorical variables.

Table 3S - Primary and secondary outcomes by intervention group and analysis populations

Outcome	Intention-to-treat set				Per-protocol set				As-treated set			
	Whole blood (n = xx)	Blood components (n = xx)	Effect estimate (95%CI)	p value*	Whole blood (n = xx)	Blood components (n = xx)	Effect estimate (95%CI)	p value*	Whole blood (n = xx)	Blood components (n = xx)	Effect estimate (95%CI)	p value*
Primary outcome												
Hierarchical composite: mortality (28d) + organ dysfunction (SOFA)†			WR: x.xx (x.xx - x.xx)	x.xxx			WR: x.xx (x.xx - x.xx)	x.xxx			WR: x.xx (x.xx - x.xx)	x.xxx
└ Sensitivity: max SOFA (days 1 - 5)‡			*WR: x.xx (x.xx - x.xx)*	*x.xxx*			*WR: x.xx (x.xx - x.xx)*	*x.xxx*			*WR: x.xx (x.xx - x.xx)*	*x.xxx*
└ Sensitivity: win odds ¶¶			*WO: x.xx (x.xx - x.xx)*	*x.xxx*			*WO: x.xx (x.xx - x.xx)*	*x.xxx*			*WO: x.xx (x.xx - x.xx)*	*x.xxx*
Secondary outcomes - mortality												
All-cause mortality at 6 hours	xx (xx)	xx (xx)	RR: x.xx (x.xx - x.xx)	x.xxx	xx (xx)	xx (xx)	RR: x.xx (x.xx - x.xx)	x.xxx	xx (xx)	xx (xx)	RR: x.xx (x.xx - x.xx)	x.xxx
All-cause mortality at 24 hours	xx (xx)	xx (xx)	RR: x.xx (x.xx - x.xx)	x.xxx	xx (xx)	xx (xx)	RR: x.xx (x.xx - x.xx)	x.xxx	xx (xx)	xx (xx)	RR: x.xx (x.xx - x.xx)	x.xxx
All-cause mortality at 28 days	xx (xx)	xx (xx)	RR: x.xx (x.xx - x.xx)	x.xxx	xx (xx)	xx (xx)	RR: x.xx (x.xx - x.xx)	x.xxx	xx (xx)	xx (xx)	RR: x.xx (x.xx - x.xx)	x.xxx
Secondary outcomes - organ dysfunction												
MOD incidence, days 1 - 5§	xx (xx)	xx (xx)	RR: x.xx (x.xx - x.xx)	x.xxx	xx (xx)	xx (xx)	RR: x.xx (x.xx - x.xx)	x.xxx	xx (xx)	xx (xx)	RR: x.xx (x.xx - x.xx)	x.xxx
Highest SOFA score, days 1 - 5	xx (xx)	xx (xx)	MD: x.xx (x.xx - x.xx)	x.xxx	xx (xx)	xx (xx)	MD: x.xx (x.xx - x.xx)	x.xxx	xx (xx)	xx (xx)	MD: x.xx (x.xx - x.xx)	x.xxx
SOFA score, day 1	xx (xx)	xx (xx)	MD: x.xx (x.xx - x.xx)	x.xxx	xx (xx)	xx (xx)	MD: x.xx (x.xx - x.xx)	x.xxx	xx (xx)	xx (xx)	MD: x.xx (x.xx - x.xx)	x.xxx
SOFA score, day 5	xx (xx)	xx (xx)	MD: x.xx (x.xx - x.xx)	x.xxx	xx (xx)	xx (xx)	MD: x.xx (x.xx - x.xx)	x.xxx	xx (xx)	xx (xx)	MD: x.xx (x.xx - x.xx)	x.xxx
ΔSOFA (day 5 - day 1)	xx (xx)	xx (xx)	MD: x.xx (x.xx - x.xx)	x.xxx	xx (xx)	xx (xx)	MD: x.xx (x.xx - x.xx)	x.xxx	xx (xx)	xx (xx)	MD: x.xx (x.xx - x.xx)	x.xxx
Secondary outcomes - SOFA subscores												
ΔSOFA respiratory (day 5 - day 1)	xx (xx)	xx (xx)	MD: x.xx (x.xx–x.xx)	x.xxx	xx (xx)	xx (xx)	MD: x.xx (x.xx–x.xx)	x.xxx	xx (xx)	xx (xx)	MD: x.xx (x.xx–x.xx)	x.xxx
Maximum SOFA respiratory, days 1 - 5	xx (xx)	xx (xx)	MD: x.xx (x.xx–x.xx)	x.xxx	xx (xx)	xx (xx)	MD: x.xx (x.xx–x.xx)	x.xxx	xx (xx)	xx (xx)	MD: x.xx (x.xx–x.xx)	x.xxx
ΔSOFA cardiovascular (day 5 - day 1)	xx (xx)	xx (xx)	MD: x.xx (x.xx–x.xx)	x.xxx	xx (xx)	xx (xx)	MD: x.xx (x.xx–x.xx)	x.xxx	xx (xx)	xx (xx)	MD: x.xx (x.xx–x.xx)	x.xxx
Maximum SOFA cardiovascular, days 1 - 5	xx (xx)	xx (xx)	MD: x.xx (x.xx–x.xx)	x.xxx	xx (xx)	xx (xx)	MD: x.xx (x.xx–x.xx)	x.xxx	xx (xx)	xx (xx)	MD: x.xx (x.xx–x.xx)	x.xxx
ΔSOFA neurological (day 5 - day 1)	xx (xx)	xx (xx)	MD: x.xx (x.xx–x.xx)	x.xxx	xx (xx)	xx (xx)	MD: x.xx (x.xx–x.xx)	x.xxx	xx (xx)	xx (xx)	MD: x.xx (x.xx–x.xx)	x.xxx
Maximum SOFA neurological, days 1 - 5	xx (xx)	xx (xx)	MD: x.xx (x.xx–x.xx)	x.xxx	xx (xx)	xx (xx)	MD: x.xx (x.xx–x.xx)	x.xxx	xx (xx)	xx (xx)	MD: x.xx (x.xx–x.xx)	x.xxx
ΔSOFA renal (day 5 - day 1)	xx (xx)	xx (xx)	MD: x.xx (x.xx–x.xx)	x.xxx	xx (xx)	xx (xx)	MD: x.xx (x.xx–x.xx)	x.xxx	xx (xx)	xx (xx)	MD: x.xx (x.xx–x.xx)	x.xxx
Maximum SOFA renal, days 1 - 5	xx (xx)	xx (xx)	MD: x.xx (x.xx–x.xx)	x.xxx	xx (xx)	xx (xx)	MD: x.xx (x.xx–x.xx)	x.xxx	xx (xx)	xx (xx)	MD: x.xx (x.xx–x.xx)	x.xxx

Continue...

...continuation

ΔSOFA coagulation (day 5 - day 1)	xx (xx)	xx (xx)	MD: x.xx (x.xx–x.xx)	x.xxx	xx (xx)	xx (xx)	MD: x.xx (x.xx–x.xx)	x.xxx	xx (xx)	xx (xx)	MD: x.xx (x.xx–x.xx)	x.xxx
Maximum SOFA coagulation, days 1 - 5	xx (xx)	xx (xx)	MD: x.xx (x.xx–x.xx)	x.xxx	xx (xx)	xx (xx)	MD: x.xx (x.xx–x.xx)	x.xxx	xx (xx)	xx (xx)	MD: x.xx (x.xx–x.xx)	x.xxx
ΔSOFA hepatic (day 5 - day 1)	xx (xx)	xx (xx)	MD: x.xx (x.xx–x.xx)	x.xxx	xx (xx)	xx (xx)	MD: x.xx (x.xx–x.xx)	x.xxx	xx (xx)	xx (xx)	MD: x.xx (x.xx–x.xx)	x.xxx
Maximum SOFA hepatic, days 1 - 5	xx (xx)	xx (xx)	MD: x.xx (x.xx–x.xx)	x.xxx	xx (xx)	xx (xx)	MD: x.xx (x.xx–x.xx)	x.xxx	xx (xx)	xx (xx)	MD: x.xx (x.xx–x.xx)	x.xxx
Secondary outcomes - coagulopathy												
Coagulopathy prevalence at 6 hours	xx (xx)	xx (xx)	RR: x.xx (x.xx - x.xx)	x.xxx	xx (xx)	xx (xx)	RR: x.xx (x.xx - x.xx)	x.xxx	xx (xx)	xx (xx)	RR: x.xx (x.xx - x.xx)	x.xxx
Coagulopathy prevalence at 12 hours	xx (xx)	xx (xx)	RR: x.xx (x.xx - x.xx)	x.xxx	xx (xx)	xx (xx)	RR: x.xx (x.xx - x.xx)	x.xxx	xx (xx)	xx (xx)	RR: x.xx (x.xx - x.xx)	x.xxx
Incident coagulopathy at 24 hours¶	xx (xx)	xx (xx)	RR: x.xx (x.xx - x.xx)	x.xxx	xx (xx)	xx (xx)	RR: x.xx (x.xx - x.xx)	x.xxx	xx (xx)	xx (xx)	RR: x.xx (x.xx - x.xx)	x.xxx
Secondary outcomes - SAE & resources												
Any serious adverse event	xx (xx)	xx (xx)	RR: x.xx (x.xx - x.xx)	x.xxx	xx (xx)	xx (xx)	RR: x.xx (x.xx - x.xx)	x.xxx	xx (xx)	xx (xx)	RR: x.xx (x.xx - x.xx)	x.xxx
Transfusion units (WB equivalents), 24 hours	xx (xx)	xx (xx)	MD: x.xx (x.xx - x.xx)	x.xxx	xx (xx)	xx (xx)	MD: x.xx (x.xx - x.xx)	x.xxx	xx (xx)	xx (xx)	MD: x.xx (x.xx - x.xx)	x.xxx
Hospital-free days at 28 days	xx (xx)	xx (xx)	MD: x.xx (x.xx - x.xx)	x.xxx	xx (xx)	xx (xx)	MD: x.xx (x.xx - x.xx)	x.xxx	xx (xx)	xx (xx)	MD: x.xx (x.xx - x.xx)	x.xxx
Tertiary outcomes (exploratory - no multiplicity adjustment)												
Vasopressor-free days at 28 days	xx (xx)	xx (xx)	-	-	xx (xx)	xx (xx)	-	-	xx (xx)	xx (xx)	-	-
Ventilator-free days at 28 days	xx (xx)	xx (xx)	-	-	xx (xx)	xx (xx)	-	-	xx (xx)	xx (xx)	-	-
RRT-free days at 28 days	xx (xx)	xx (xx)	-	-	xx (xx)	xx (xx)	-	-	xx (xx)	xx (xx)	-	-
Damage control surgery, n (%)	xx (xx)	xx (xx)	-	-	xx (xx)	xx (xx)	-	-	xx (xx)	xx (xx)	-	-
Resuscitation fluid volume, 24 hours (mL)	xx (xx)	xx (xx)	-	-	xx (xx)	xx (xx)	-	-	xx (xx)	xx (xx)	-	-
Serum lactate at 3, 6, 12, 24 h (mmol/L)	xx (xx)	xx (xx)	-	-	xx (xx)	xx (xx)	-	-	xx (xx)	xx (xx)	-	-

SOFA - Sequential Organ Failure Assessment; MOD - multiple organ dysfunction; SAE - serious adverse event; WB - whole blood; RRT - renal replacement therapy. * Two-tailed p-value; Mann-Whitney U test for quantitative variables, chi² for categorical variables. Significance threshold α=0.05. No multiplicity adjustment applied to secondary outcomes; interpret in context of overall pattern of results; † primary outcome: hierarchical composite of all-cause mortality at 28 days (Level 1) and SOFA score change from day 1 to day 5 (Level 2); analyzed using win-ratio method. WR > 1 favors Treatment A (whole blood); ‡ pre-specified sensitivity analysis: Level 2 replaced by maximum SOFA score recorded on any day from day 1 through day 5. Lower maximum SOFA score = win for that participant's group; ¶¶ pre-specified sensitivity analysis: win odds estimated using the WINS package (method = "win odds"), incorporating ties into both numerator and denominator. All other analytical specifications remain identical to the primary analysis. The 95% CI is constructed on the log scale using a U-statistic-based variance estimator accounting for the covariance among wins, losses, and ties, then back-transformed. § multiple organ dysfunction defined as SOFA ≥ 2 points in one organ system or ≥ 1 point in two or more systems on the same day; ¶ incident coagulopathy at 24 h: patients with coagulopathy present at baseline excluded from denominator; - tertiary/exploratory outcomes: effect estimates and p-values not pre-specified; reported descriptively only. Data are presented as n (%) for categorical variables and median (interquartile range) for continuous variables, unless otherwise specified.

Table 4S - Adverse events by category, intervention group and analysis populations

Adverse event	Intention-to-treat set			Per-protocol set			As-treated set		
	Whole blood (n = xx)	Blood components (n = xx)	p value*	Whole blood (n = xx)	Blood components (n = xx)	p value*	Whole blood (n = xx)	Blood components (n = xx)	p value*
Non-serious adverse events									
Any non-serious adverse event	xx (xx)	xx (xx)	x.xxx	xx (xx)	xx (xx)	x.xxx	xx (xx)	xx (xx)	x.xxx
Non-hemolytic febrile reaction	xx (xx)	xx (xx)	-	xx (xx)	xx (xx)	-	xx (xx)	xx (xx)	-
Allergic reaction (non-anaphylactic)	xx (xx)	xx (xx)	-	xx (xx)	xx (xx)	-	xx (xx)	xx (xx)	-
Potassium disorders	xx (xx)	xx (xx)	-	xx (xx)	xx (xx)	-	xx (xx)	xx (xx)	-
Calcium disorders	xx (xx)	xx (xx)	-	xx (xx)	xx (xx)	-	xx (xx)	xx (xx)	-
SAE†									
Any SAE	xx (xx)	xx (xx)	x.xxx	xx (xx)	xx (xx)	x.xxx	xx (xx)	xx (xx)	x.xxx
Any SAE adjudicated as related‡	xx (xx)	xx (xx)	x.xxx	xx (xx)	xx (xx)	x.xxx	xx (xx)	xx (xx)	x.xxx
Anaphylaxis	xx (xx)	xx (xx)	-	xx (xx)	xx (xx)	-	xx (xx)	xx (xx)	-
Acute hemolytic reaction	xx (xx)	xx (xx)	-	xx (xx)	xx (xx)	-	xx (xx)	xx (xx)	-
Acute lung injury (ALI)	xx (xx)	xx (xx)	-	xx (xx)	xx (xx)	-	xx (xx)	xx (xx)	-
Adult respiratory distress syndrome (ARDS)	xx (xx)	xx (xx)	-	xx (xx)	xx (xx)	-	xx (xx)	xx (xx)	-
Acute kidney injury (AKI)	xx (xx)	xx (xx)	-	xx (xx)	xx (xx)	-	xx (xx)	xx (xx)	-
Pulmonary thromboembolism	xx (xx)	xx (xx)	-	xx (xx)	xx (xx)	-	xx (xx)	xx (xx)	-
Deep venous thrombosis	xx (xx)	xx (xx)	-	xx (xx)	xx (xx)	-	xx (xx)	xx (xx)	-
Ventilator-associated pneumonia	xx (xx)	xx (xx)	-	xx (xx)	xx (xx)	-	xx (xx)	xx (xx)	-
Urinary tract infection	xx (xx)	xx (xx)	-	xx (xx)	xx (xx)	-	xx (xx)	xx (xx)	-
Surgical site infection (superficial)	xx (xx)	xx (xx)	-	xx (xx)	xx (xx)	-	xx (xx)	xx (xx)	-
Surgical site infection (deep)	xx (xx)	xx (xx)	-	xx (xx)	xx (xx)	-	xx (xx)	xx (xx)	-
Sepsis	xx (xx)	xx (xx)	-	xx (xx)	xx (xx)	-	xx (xx)	xx (xx)	-
Septic shock	xx (xx)	xx (xx)	-	xx (xx)	xx (xx)	-	xx (xx)	xx (xx)	-
Abdominal complications	xx (xx)	xx (xx)	-	xx (xx)	xx (xx)	-	xx (xx)	xx (xx)	-
Abdominal hypertension syndrome	xx (xx)	xx (xx)	-	xx (xx)	xx (xx)	-	xx (xx)	xx (xx)	-
Upper digestive bleeding	xx (xx)	xx (xx)	-	xx (xx)	xx (xx)	-	xx (xx)	xx (xx)	-
Stroke	xx (xx)	xx (xx)	-	xx (xx)	xx (xx)	-	xx (xx)	xx (xx)	-
Acute myocardial infarction	xx (xx)	xx (xx)	-	xx (xx)	xx (xx)	-	xx (xx)	xx (xx)	-

SAE - serious adverse event; ALI - acute lung injury; ARDS - adult respiratory distress syndrome; AKI - acute kidney injury. * Two-tailed p-value from chi² test for aggregate rows (any non-serious adverse event, any serious adverse event, any related serious adverse event). Individual event rows are underpowered for formal testing and are reported descriptively (-). If absolute mortality difference between groups exceeds 5%, competing risks analysis (subdistribution hazard model) will replace chi² for cumulative incidence outcomes; † serious adverse events defined per Good Clinical Practice Guidelines (ICH E6). Anaphylaxis and acute hemolytic reaction are classified as serious adverse events given their potential to be life-threatening, regardless of actual outcome; ‡ causality adjudicated by the independent adjudication committee; 'related' includes 'possibly related' and 'probably related' categories. Data are presented as n (%).
